# Utilization of health services among the elderly in Iran during the COVID‐19 outbreak: A cross‐sectional study

**DOI:** 10.1002/hsr2.839

**Published:** 2022-09-21

**Authors:** Farzad Faraji‐Khiavi, Habib Jalilian, Somayeh Heydari, Reza Sadeghi, Morteza Saduqi, Seyed‐Ali Razavinasab, Majid Heidari‐Jamebozorgi

**Affiliations:** ^1^ Department of Health Services Management, School of Health Ahvaz Jundishapur University of Medical Sciences Ahvaz Iran; ^2^ Department of Public Health Sirjan School of Medical Sciences Sirjan Iran; ^3^ Department of Laboratory Sciences Sirjan School of Medical Sciences Sirjan Iran; ^4^ Imam Reza Hospital Sirjan School of Medical Sciences Sirjan Iran; ^5^ Department of Public Health Sirjan School of Medical Sciences Sirjan Iran

**Keywords:** COVID‐19, elderly, healthcare access, healthcare utilization, health services utilization

## Abstract

**Background and Aims:**

Elderly people are potentially vulnerable with a higher need for health services, and utilization of Essential Public Health Services (EPHS) among this group is of high importance. This study aimed to examine the utilization of health services among the elderly in Iran during the coronavirus disease 2019 outbreak.

**Methods:**

This was a cross‐sectional study conducted in 21 public health centers in Sirjan, Southern Iran, from May to December 2020. A total of 420 elderly patients were selected through a systematic random sampling method. Data were collected using a questionnaire and were analyzed using SPSS _v22.0_. The binary logistic regression was used to examine the effect of demographic, socioeconomic and morbidity status on inpatient and outpatient healthcare utilization.

**Results:**

Our results showed that 56% of the elderly had a history of hospitalization during the last year. Although 60% of the elderly reported they had a perceived need for outpatient services, only 49% of them reported that they utilized outpatient services. 51% and 35.5% of the elderly reported that their inpatient and outpatient costs were covered by health insurance, respectively. Others reported their health spending was financed through out‐of‐pocket payments. Male gender aged 80 and above, urban residents, higher socioeconomic and supplemental insurance coverage were associated with an increase in health services utilization. The elderly with Cancer, mental disorders, kidney disease, and cardiovascular diseases (CVDs) were more likely to be hospitalized.

**Conclusion:**

There were demographic and socioeconomic inequalities in health services utilization among the elderly. Therefore, appropriate interventions and strategies are needed to reduce these inequalities in health services utilization among the elderly. In addition, given that the hospitalization rate was significantly higher among the elderly with chronic diseases than those without, it is crucial and necessary to take interventions to reduce the burden of chronic diseases in the future.

## 1 BACKGROUND

The elderly population is on the rise rapidly worldwide, with the world gaining one million older persons each month.^1^ According to World Health Organization (WHO) report, the proportion of the world's population over 60 years of age will nearly double, from 12% to 22%, between 2015 and 2050.^2^ Like other countries in the world, Iran is facing an aging population. Only 8% of the Iranian population was 60 or above in 2015. It is projected this figure to reach 11% in 2025 and about 33% of the total population in 2050.^3^

Many countries are facing with challenges of the aging population, especially in the domain of health.^4^ The aging population will increase the prevalence of Noncommunicable diseases (NCDs) and the demand for healthcare services, which puts new strains on healthcare provision in the future.^5^, ^6^, ^7^, ^8^

Access to and utilization of health services are two key factors to improving health outcomes, especially in Low‐ and Middle‐income Countries (LMICs).^9^ The utilization of essential public health services (EPHS) among old‐aged people is a challenging issue especially for developing countries to achieve universal health coverage (UHC).^10^, ^11^, ^12^

The coronavirus disease 2019 (COVID‐19) pandemic has had a major impact on the global health system. When hospitals and private practices attempted to respond to COVID‐19 emergencies, routine and elective services and procedures tended to be reduced to a minimum level, with unavoidable consequences for chronic disease patients' health care.^13^ Previous studies demonstrated that healthcare visits such as inpatient visits, emergency department (ED) visits, and outpatient visits have substantially decreased since the start of the Covid‐19.^14^, ^15^, ^16^, ^17^ Moynihan et al. examined the health system response to COVID‐19 in 20 countries and estimated that healthcare utilization fell by more than one‐third during the pandemic (37%).^18^

The coincidence of the COVID‐19 pandemic and its outcomes in Iran with the highest unilateral sanctions imposed by the United States against Iran has posed many obstacles to the country's health sector.^19^ A recently previous study in Iran demonstrated that COVID‐19 had a negative consequence on the utilization of services. The percentage of actually delivered services and also the weighted average of essential services provided by a physician, dentist, midwife, mental health experts, and nutritionist decreased after the occurrence of the COVID‐19 pandemic.^20^

In the context of health, knowing and understanding how people utilize health care is critical for allocating resources and planning. However, one of the key priorities of health policymakers is to reduce health inequalities and access to health services among different groups, especially elderly ones. By examining the status of health‐care utilization among the elderly, we can obtain a clear picture of the demand of the elderly population for health services, understand how to respond to this demand, allocate resources, and how to plan to provide health services in the future. Therefore, this study aimed to survey the status of health services utilization (inpatient and outpatient services utilization, the most important reason for the use of the services, factors influencing services utilization, and type of the centers where the elderly were referred to receive services) and the associated factors among the elderly. Our findings can provide useful information and assist health system managers and health policymakers to plan to fund required resources in the future and provide the elderly population with better service provision.

## 2 METHOD AND MATERIALS

### 2.1 Study design and setting

This cross‐sectional study was conducted in public health centers affiliated with Sirjan School of Medical Sciences from May to December 2020 in Sirjan city, southern Iran.

### 2.2 Study participants and sampling

The study population includes all adults aged 60 and over who are registered in the National Integrated Health System (SIB). Sirjan Faculty of Medical Sciences includes 21 urban and rural health centers. All centers were selected for sampling. Sampling was done by systematic random method from these health centers. Due to the fact that these centers cover all areas of the city and cover the same number of people. 20 elderly people were assigned to each center. According to the division of the source population by the sample size, the sampling distance *k* = 4 was calculated and this distance was used in all health centers to select the participants. The first subject was selected by simple random sampling. The sample size was estimated based on a previous study,^21^, ^22^ considering a possible prevalence of 50%, 5% margin of error (d), a *Z*‐value of 1.96, 95% confidence interval, and a design effect of 1.2. The sample size was calculated as 420 subjects.

### 2.3 Data collection tools and techniques

A self‐reported questionnaire was used to collect data. The questionnaire consisted of 36 questions divided into three parts. The first part consisted of demographic and socioeconomic status such as age, gender, education status, marital status, income level, and insurance coverage status (13 questions). The second part consisted of questions on the status of morbidity (10 questions) and the third on the status of health services utilization (13 questions). We applied a recall time horizon of 4 weeks for outpatient service utilization and the recall time horizon of the past 12 months (period) for inpatient services utilization. In this study, first, the purpose of the study was explained to the patients or members of their families by telephone, and informed consent to participate in the study was obtained from each participant. The participants were informed that participation in this study was voluntary, and they could refuse participation without any consequences. Next, a web link to the online questionnaire was sent via WhatsApp to those who were literate. Illiterate respondents were contacted by telephone, and questions were read out by the researcher and they were asked to answer each question. The reliability and validity of the questionnaire was test by internal consistency (Cronbach's *α* = 0.86).

### 2.4 Data analysis

Data were analyzed using SPSS _v22.0_. Mean, standard deviation (SD) and frequency were used for data description. The Chi‐square test was used for analyzing relationships and performing between‐group comparisons. We used binary logistic regression to examine the effect of demographic, socioeconomic, and morbidity status on inpatient and outpatient healthcare utilization.

### 2.5 Ethics approval and consent to participate

This study was approved by the Ethics Committee of the Sirjan School of Medical Sciences (Reference No: IR.SIRUMS.REC.1399.011). All participants gave informed verbal consent before participating in the study. All the participants were given details about the purpose of the study. Furthermore, it was stated that participation was entirely voluntary and that their anonymity would be ensured. All methods were carried out in accordance with the relevant guidelines and regulations.

## 3 RESULTS

The demographic and socioeconomic profiles of participants are shown in Table 1. The majority of participants (91.2%) were either illiterate or diploma or lower. Most of the participants had low incomes. Also, 91.7% of participants were covered by basic insurance, and about half of them were covered by supplemental insurance.

**Table 1:** Demographic and socioeconomic profile of participants and their association with service utilization

Variables	Groups	Frequency (%)	Inpatient utilization			Outpatient utilization		
			Utilization %	*χ* ^2^	*p* value	Utilization %	*χ* ^2^	*p* value
Gender	Male	206 (49.0)	56.8	0.11	0.73	28.6	0.06	0.81
	Female	214 (51.0)	55.1			29.7		
Age	60–70	218 (53.8)	52.8	3.06	0.21	26.3	4.93	0.08
	70–80	134 (33.1)	61.9			31.6		
	> 80	53 (13.1)	52.8			41.5		
Education status	Illiterate	166 (39.4)	62	4.93	0.08	36.6	7.89	0.02*
	Diploma or lower	218 (51.8)	50.9			25.2		
	Academic education	37 (8.8)	59.5			18.9		
Employment status	Employed	252 (59.9)	53.6	1.57	0.21	28	0.37	0.54
	Retired	169 (40.1)	59.8			30.8		
Marital status	Single	104 (24.8)	53.8	0.31	0.58	33.7	1.33	0.25
	Married	316 (75.2)	57			27.7		
Residence status	Urban	338 (80.3)	69.9	8.02	<0.001**	31.5	4.85	0.02*
	Rural	83 (19.7)	52.7			19.3		
Income status(Month)	Low income	151 (36.2)	62.9	11.9	<0.001**	35.6	5.77	0.12
	Under middle income	145 (34.8)	53.8			28.3		
	Upper middle income	75 (18.0)	40			22.7		
	High income	46 (11.0)	63			21.7		
Basic health insurance status	Yes	386 (91.7)	56	0.02	0.89	28.6	0.49	0.48
	No	35 (8.3)	57.1			34.3		
Type of basic health insurance	Social security	224 (54.4)	55.4	0.85	0.65	29.5	0.31	0.85
	Iranian health	82 (19.9)	54.9			26.3		
	Others	106 (25.7)	60.4			29.2		
Supplementary health insurance status	Yes	213 (50.6)	55.9	0.00	0.93	30	0.81	0.67
	No	208 (49.4)	56.3			28.2		
Smoking	Yes	87 (20.7)	55.2	0.035	0.85	33.3	0.94	0.33
	No	334 (79.3)	56.3			28		

* Significant at *p* < 0.05 level.

** Significant at *p* < 0.01 level.

**Figure 1:**
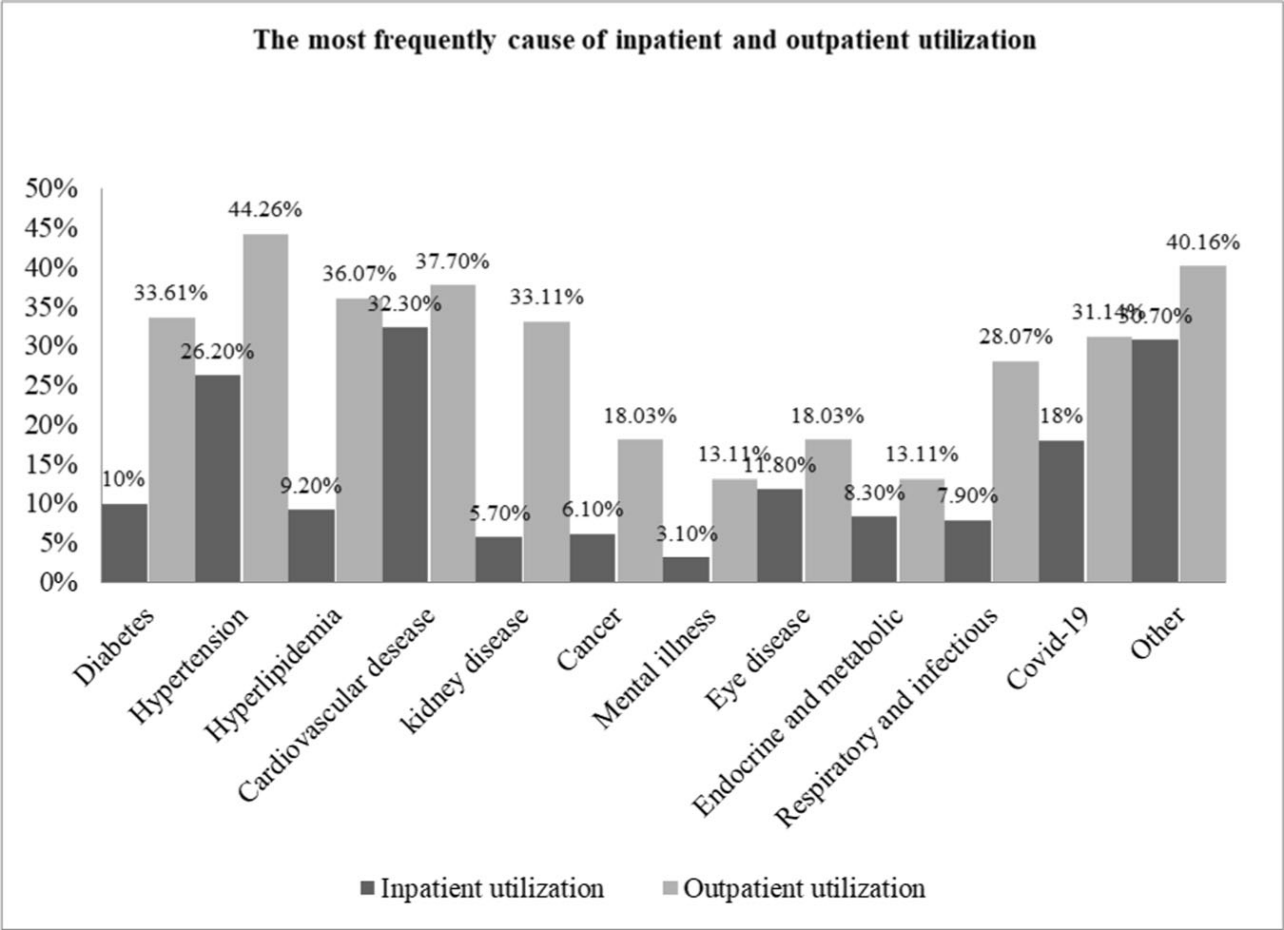
The most frequent cause of service utilization in elderly.

Our results showed that 56% of the elderly had a history of hospitalization during the last year. Concerning outpatient services, although 60% of the elderly reported they had a perceived need for outpatient services, only 49% of them reported that they utilized outpatient services. Figure 1 presents the most frequent cause of inpatient and outpatient utilization in the elderly. Cardiovascular diseases (CVDs) (32.30%), hypertension (26.20%), and COVID‐19 (18%) were the most common cause of hospitalization, respectively during last year. Furthermore, hypertension (44.26%), CVDs (37.70%), hyperlipidemia (36.07%), and kidney disease (33.11%) were, respectively, the most common reason for outpatient services utilization during the past month.

**Figure 2:**
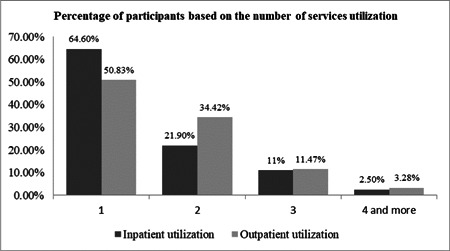
The percentage of participants based on the number of services utilization.

**Figure 3:**
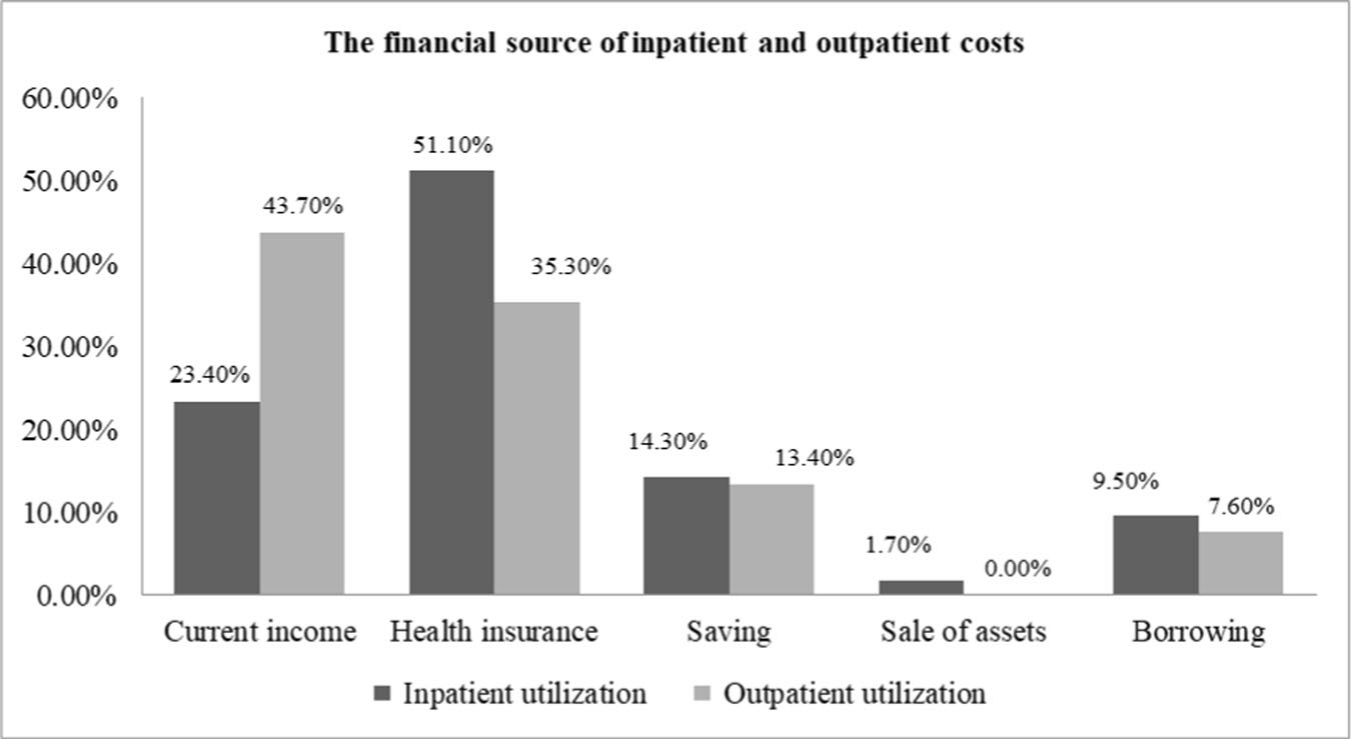
The financial source of inpatient and outpatient costs.

**Figure 4:**
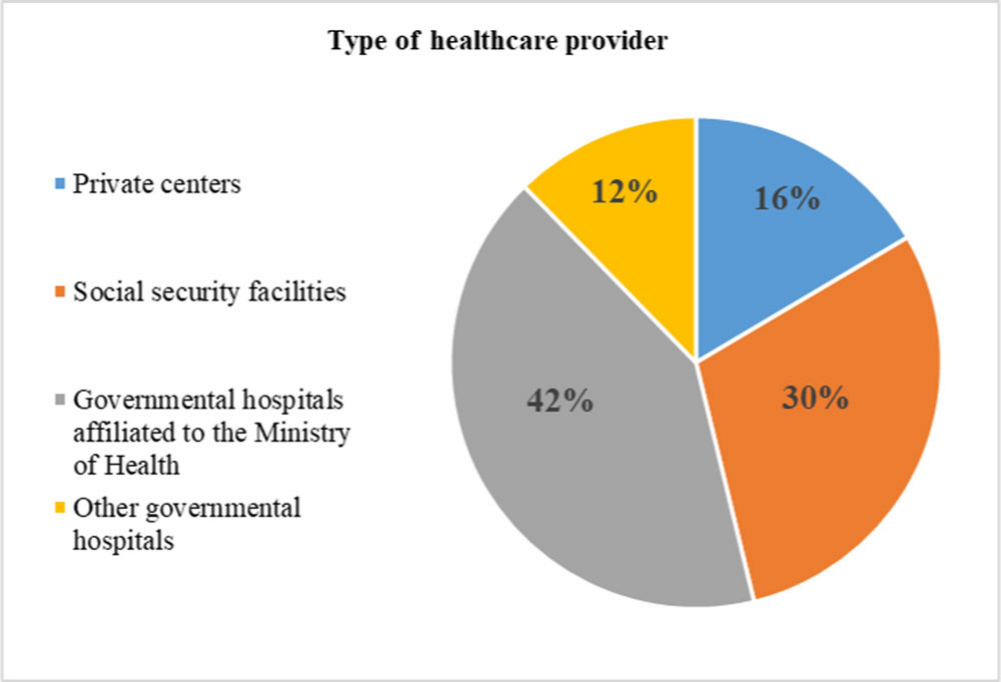
Type of healthcare provider that patients referred to receive services.

In this study, 51% and 35.5% of the elderly reported that their inpatient and outpatient costs were paid by health insurance, respectively. Others reported that to finance their health spending they had to use their current income and savings, or had to sell assets/borrow from others (Figures 2 and 3). The majority of the elderly reported receiving health services from governmental hospitals affiliated with the Ministry of Health (41.5%) (Figure 4). The results of the Chi‐squared test showed a significant association between inpatient utilization and residence status and income level (*p* < 0.05). Those residing in urban areas and those with higher incomes were more likely to be hospitalized. Furthermore, a significant association was found between outpatient utilization and residence status and education (*p* < 0.05). Those residing in urban areas and those with lower education were more likely to utilize outpatient services.

The morbidities and their association with healthcare utilization are shown in Table 2. Hypertension, hyperlipidemia, and CVDs increased significantly the probability of both inpatient and outpatient healthcare utilization (*p* < 0.05). Kidney disease, cancer, and mental disorders were significantly associated with inpatient services utilization. Diabetes, endocrine, and metabolic, and respiratory and infectious diseases were significantly associated with outpatient service utilization (*p* < 0.05). Those with these diseases were more likely to utilize healthcare services than those without. According to the most common reasons for utilizing from inpatient and outpatient services among comorbidity diseases, except for kidney diseases, other factors had a significant relationship (Table 2, Figure 1).

**Table 2:** Prevalence of morbidities and their association with service utilization

Morbidity status	Groups	Frequency (%)	Inpatient utilization			Outpatient utilization		
			Utilization%	*χ* ^2^	*p* value	Utilization%	*χ* ^2^	*p* value
Diabetes	Yes	117 (27.9)	60.7	1.33	0.25	37.6	5.57	0.01*
	No	303 (72.1)	54.5			25.9		
Hypertension	Yes	234 (55.6)	60.7	4.57	0.03*	34.1	6.13	0.01*
	No	187 (44.4)	50.3			23		
Hyperlipidemia	Yes	167 (39.8)	67.1	13.89	<0.001**	42.4	24.07	<0.001**
	No	253 (60.2)	48.6			20.2		
Cardiovascular diseases	Yes	116 (27.6)	75	23.6	<0.001**	38.3	6.32	0.01*
	No	304 (72.4)	48.7			25.7		
Kidney disease	Yes	40 (9.5)	77.5	9.25	<0.001**	40	2.53	0.11
	No	381 (90.5)	53.8			28		
Cancer	Yes	19 (4.5)	89.5	9.02	<0.001**	42.1	1.62	0.20
	No	402 (95.5)	54.5			28.5		
Mental disease	Yes	36 (8.6)	75	5.73	0.01*	34.3	0.49	0.48
	No	385 (91.4)	54.3			28.6		
Eye disease	Yes	145 (34.6)	62.1	3.48	0.06	29.2	0.01	0.90
	No	274 (65.4)	52.6			28.6		
Endocrine and metabolic	Yes	68 (16.2)	55.9	0.001	0.99	44.1	8.76	<0.001*
	No	352 (83.8)	56.1			26.3		
Respiratory and infectious	Yes	68 (16.2)	64.7	2.4	0.12	48.5	14.7	<0.001**
	No	352 (83.8)	54.5			25.4		

* Significant at *p* < 0.05 level.

** Significant at *p* < 0.001 level.

Table 3 reports the results of the Binary Logistic Regression model. The omnibus test results (*p* < 0.05) indicates that the current model outperforms the null model.

**Table 3:** Binary logistic regression model for factors affecting inpatient utilization

Parameter	*B*	SE	Exp (*B*)	95% CI for EXP (*B*)		*p* value
				Lower	Upper	
Gender (reference group = female)						
Male	−0.20	0.28	0.81	0.46	1.42	0.46
Marital status (reference group = married)						
Single	0.36	0.28	1.44	0.82	2.51	0.20
Residence (reference group = rural)						
Urban	1.27	0.37	3.57	1.70	7.50	<0.001*
Income (reference group = $300 and more)						
Less than $100	0.49	0.50	1.63	0.60	4.40	0.33
$100 to $200	1.03	0.48	2.80	1.08	7.23	0.03*
$200 to $300	1.44	0.49	4.24	1.60	11.20	<0.001*
Basic insurance (reference group = no)						
Yes	0.18	0.57	1.19	0.39	3.68	0.75
Supplementary insurance (reference group = no)						
Yes	0.21	0.26	1.23	0.73	2.08	0.42
Age (reference group => 80)						
60‐70	−0.73	0.42	0.48	0.21	1.10	0.08
70‐80	−1.01	0.42	0.36	0.15	0.83	0.02*
Education (reference group = academic education)						
Illiterate	0.32	0.57	1.37	0.44	4.25	0.57
Diploma or lower	0.49	0.49	1.648	0.623	4.36	0.31
Employment status (reference group = retired)						
Employed	0.62	0.29	1.858	1.04	3.29	0.03*
Basic insurance type (reference group = other)						
Social security	0.41	0.29	1.50	0.84	2.69	0.16
Iranian health	1.15	0.43	3.15	1.34	7.44	0.01*
Diabetes (reference group = no)						
Yes	0.18	0.29	1.20	0.67	2.13	0.53
Hypertension (reference group = no)						
Yes	0.49	0.27	1.63	0.96	2.78	0.06
Hyperlipidemia (reference group = no)						
Yes	0.42	0.28	1.53	0.87	2.67	0.13
Cardiovascular diseases (reference group = no)						
Yes	1.25	0.28	3.50	1.99	6.15	<0.001*
Kidney disease (reference group = no)						
Yes	1.28	0.50	3.59	1.33	9.67	0.01*
Cancer disease (reference group = no)						
Yes	2.04	0.80	7.70	1.58	37.54	0.01*
Mental disease (reference group = no)						
Yes	1.49	0.50	4.43	1.662	11.846	<0.001*
Eye disease (reference group = no)						
Yes	0.22	0.26	1.25	0.74	2.10	0.39
Endocrine disease (reference group = no)						
Yes	0.01	0.35	1.01	0.50	2.01	0.99
Infectious and respiratory diseases (reference group = no)						
Yes	−0.20	0.35	0.82	0.41	1.64	0.57

Abbreviations: CI, confidence interval; SE, standard error.

* *p* < 0.05 was considered as significant.

Nagelkerke R Square was estimated at 0.3, so model explains 30% of variation outcome. Hosmer and Lemeshow Test was estimated at 0.8. However, the Likelihood function is approved and shows that the model fits the data well. The results showed that those who were aged 70–80 years were 0.64 less likely to be hospitalized than those aged >80 (*p* < 0.05). Those living in urban areas were almost 3.57 times more likely to be hospitalized than those living in rural areas (*p* = 0.001). The probability of hospitalization was higher among those with moderate‐income and employed ones (*p* < 0.05). Those who were under the coverage of Iranian health insurance were 3.15 times more likely to be hospitalized during the last year (*p* < 0.05). Furthermore, those with cancer (7.7 times), mental disorders (4.43 times), kidney disease (3.59 times), and CVDs (3.5 times) were more likely to be hospitalized during the last year (*p* < 0.05).

## 4 DISCUSSION

This study aimed to examine the utilization of health services among the elderly in the south of Iran during the COVID‐19 outbreak. In the present study, more than half of the elderly stated they had a history of hospitalization during the last year. Although 60% of the elderly had perceived a need for outpatient services, only 49% of them stated they utilized these services during the past month. This can be attributed in part to socioeconomic barriers, especially financial barriers,^23^ and in part to the fear of being infected with COVID‐19 and cancellation of appointments either by a healthcare provider or patient during the pandemic.^24^ Studies in most countries showed a significant decrease in the use of different health services during the COVID‐19 pandemic.^24^, ^25^, ^26^, ^27^, ^28^, ^29^, ^30^, ^31^, ^32^, ^33^, ^34^ Sing and colleagues conducted a qualitative study to examine community perceptions of COVID‐19 and their experiences towards health services utilization during the pandemic in Nepal. Their study showed that some factors such as public fear and anxiety of COVID‐19, transportation disruptions, unavailability of medicines and health services at local health facilities, poor management of quarantine, isolation and testing facilities of COVID‐19, and limited involvement of private healthcare sectors were major barriers in health services utilization during COVID‐19 pandemic.^35^

More importantly, our study revealed that, although the majority of patients were covered by basic health insurance and nearly half of them were covered by supplemental health insurance, approximately half of the elderly had to dip into their savings, use their current income, borrow money from others, or sell their assets to pay their treatment costs. This highlights that the cost and service coverage of the medical insurance system is as important as population coverage to improve the financial protection of the elderly and access to healthcare services. Therefore, more attention should be directed towards the improvement of service coverage and

reimbursement schemes for elderly people. Given that, elderly people have a higher need for health services, it is essential for health insurance organizations to increase their support in these vulnerable groups. Our results are consistent with the findings of a study in India that examined the utilization and financing of elderly inpatient care to unravel intersecting inequalities in distressed financing. The authors demonstrated that households are more likely to resort to means such as borrowings, asset selling and contributions from friends and relatives to support the hospitalization of elderly males.^36^ Gupta and colleagues have estimated healthcare utilization and expenditure patterns for cardio‐metabolic diseases in South Asian cities and showed that most of the patients for paying their treatment expenditures had to use their own savings or borrow from family members, and only a small part of expenditures was paid by insurance.^37^, ^38^ In a study in China, the hospitalization reimbursement ratio was far below the overall level, and OOP was substantially higher than that of the Organization for Economic Co‐operation and Development (OECD) countries.^39^ It appears that in developing countries, health insurance coverage is poor.

In our study, the most frequent reasons stated for hospitalization during the last year were CVDs, COVID‐19, and hypertension, respectively. It seems that health systems around the world are over‐stressed, both due to non‐communicable diseases and new communicable ones like COVID‐19. Moreover, in the current study, hypertension, CVDs, hyperlipidemia, diabetes, and kidney disease respectively, were the most common cause of outpatient services utilization during the past month. Those with hypertension, CVDs, and COVID‐19 were more likely to utilize both inpatient and outpatient services. The results of the Chi‐square and Binary Logistic Regression model confirmed these diseases had an impact on increasing health services utilization. A previous study demonstrated that even modestly heightened blood pressure variability posed a substantial risk for hospitalization and mortality in the adult population.^40^ Another study showed that even modestly high blood pressure was associated with frequent cardiovascular events, even in well‐controlled hypertensive.^41^ A study in China demonstrated that elderly patients with NCDs used more inpatient services.^42^ Studies showed that higher functional impairment and a higher number of comorbid were significantly associated with service utilization in elderly patients.^43^, ^44^, ^45^, ^46^ Given the increasing aging population, health systems will face more difficulties due to the increasing prevalence of NCDs in the future. These diseases require regular and continuous care and impose a manifold burden on healthcare systems. Hence, to appropriately respond to these difficulties, it is necessary to take intervention to improve the lifestyle of people from birth and long‐term planning to create healthy aging, and the capacity of health systems increase to meet the growing demand. Smith and colleagues showed that regular primary care was associated with an 89% increased likelihood of blood pressure control and 177% increased likelihood of glycemic control.^47^

Moreover, those with higher incomes were more likely to be hospitalized during the last year. Household income is a factor affecting the use of inpatient services, which is an important determinant of inequality in inpatient services utilization, as more wealthy individuals can pay for and utilize more inpatient services.^48^, ^49^, ^50^ Older adults usually have more need for health care but are often less able to afford them.^51^ Several studies have shown that ailing elderly people with lower income are less likely to have access to healthcare services and vice versa.^52^, ^53^, ^54^, ^55^ A study in China showed that affordability of health care is the major barrier to healthcare utilization among the mid‐aged and elderly, and a higher share of Out‐of‐Pocket (OOP) payment was negatively correlated with healthcare utilization.^56^ Another study in China in 2020 demonstrated that the affluent middle‐aged and elderly patients used more inpatient services than poor groups. The authors suggested that per capita household consumption expenditure contributed the greatest pro‐rich inequality, which indicated that the difference in economic level was still the prime factor leading to the unfairness of inpatient use in China.^42^ More studies showed that the higher OOP payment was negatively associated with healthcare utilization and the high cost of services is a major barrier to healthcare utilization in developed and developing nations.^56^, ^57^, ^58^, ^59^

Our results indicated that the elderly aged 80 and above were more likely to be hospitalized than those aged 70–80. Our results are contrary to previous studies which found that middle‐old individuals were at higher risk of hospitalization.^42^, ^60^ Another study in the United States examined spatiotemporal and demographic trends in CV disease in the US elderly based on 108 million hospitalization records and showed that CV disease hospitalization rates increased from 1991 to 2004 for women aged 65–90 and men aged 65–80, but declined in the oldest‐old.^61^

Moreover, we found that the utilization of healthcare services was favoring those living in urban areas. This is not difficult to understand because people in urban areas due to better urban transport infrastructure and low costs of transport have better and more access to health services and also have a much higher likelihood of seeking medical treatment than those living in rural areas. Another reason might be that the rural community‐dwelling elderly persons in the country are mainly self‐employed in the agricultural sector without any retirement schemes or pension benefits, therefore due to their scanty savings, they are less likely to seek healthcare services or less tended to be hospitalized in hospital. Identifying the inequality of healthcare utilization among rural‐urban residents can assist in health policymakers adopting tailored policies to reduce such inequalities. A study in Iran showed that inequality in utilization of outpatient healthcare services increased from 0.105 to 0.133 between 2008 and 2016, and residence in rural areas and supplemental insurance were the main contributing factors in the increased inequality of utilization of outpatient healthcare services.^62^ More studies confirmed the rural–urban inequality in healthcare utilization among the elderly population and showed that the elderly in the urban had a better utilization than the elderly in the rural areas.^52^, ^63^, ^64^ A study in India showed that poor quality of rural health infrastructure coupled with inaccessibility has been found to be the major barriers to utilizing healthcare services by the tribal population.^65^

Furthermore, utilization of inpatient and outpatient services was favoring those with supplemental and Iranian health insurance. Healthcare insurance decreases the barriers for individuals to the utilization of health services,^66^, ^67^, ^68^, ^69^ and those who have health insurance have a higher rate of treatment‐seeking than those who are not insured.^52^ The government and health policymakers should redesign social healthcare insurance programs to further protect the elderly population, especially those with chronic disease. The results are consistent with previous studies that have examined the effect of health insurance on access to care and inequality in healthcare utilization,^70^, ^71^, ^72^, ^73^, ^74^, ^75^, ^76^ where health insurance was associated with greater access to healthcare services among older patients. For example, it has been shown that whereas education status and basic health insurance were the main contributing factors to inequalities in the utilization of outpatient healthcare services in 2006, both basic and complementary health insurance and educational status were next in the utilization of healthcare services in 2016.^62^ A study in China showed that the expanded medical insurance schemes in China may stimulate the healthcare‐seeking behaviors of the insured and unleash healthcare demands of the disadvantaged population in general.^77^

The population of Iran due to very rapid demographic changes in the past and a significant fertility decline along with the rise in life expectancy is rapidly aging,^78^ and also owing to changing disease risk factors is now facing NCDs as the major health problem.^79^ Given the growing aging population and the prevalence of these diseases, it seems that a double burden will impose on Iran's health system in the future. Therefore, planning for increased the capacity of service provision (e.g., more beds and manpower in health centers), and alternative mechanisms to these services such as home care and palliative care is highly necessary. Additionally, in order that younger generation to be a healthier generation and suffer fewer these diseases in the future, long‐term strategies should be taken.

## 5 LIMITATIONS

There are several limitations to be acknowledged in this study. First, this study was based on self‐reported data, which is prone to recall bias. Second, this was a cross‐sectional design, which did not allow us to assess the causality effect of the COVID‐19 epidemic on the rate of health services utilization. Therefore, further studies should assess this causality using the time series analysis method and before and after COVID‐19.

### 5.1 Policy implications

Our study provides important policy implications to improve equitable access to health care in Iran. First, since the hospitalization rate and utilization of outpatient services are significantly higher in the elderly with chronic diseases than in those without these diseases, it is necessary to strengthen the prevention and treatment of chronic diseases among these groups and elevate their health status in the coming years. Second, due to socioeconomic inequalities, appropriate interventions and strategies are needed to reduce the socioeconomic inequalities in health service utilization. Third, those with Iranian health insurance and supplemental insurance are more likely to use outpatient and inpatient services. Therefore, strategies should be taken to expand health insurance coverage in other groups.

## 6 CONCLUSION

The results from this study showed the demographic and socioeconomic inequalities in health services utilization. Health policies interventions and appropriate strategies are needed to reduce the socioeconomic inequalities in health services utilization among the elderly. In addition, given that the hospitalization rate was significantly higher among the elderly with chronic diseases than those without, it is crucial and necessary to take interventions to modify lifestyle and strengthen preventive care to reduce the burden of chronic diseases in the future.

## AUTHOR CONTRIBUTIONS

**Farzad Faraji‐Khiavi**: Writing – review & editing. **Habib Jalilian**: Conceptualization; data curation; formal analysis; methodology; software; validation; writing – original draft; writing – review & editing. **Somayeh Heydari**: Writing – review & editing. **Reza Sadeghi**: Writing – review & editing. **Morteza Saduqi**: Data curation. **Seyed‐Ali Razavinasab**: Writing – review & editing. **Majid Heidari‐Jamebozorgi**: Data curation; Funding acquisition.

## CONFLICTS OF INTEREST

The authors declare no conflicts of interest.

## TRANSPARENCY STATEMENT

The lead author Majid Heidari‐Jamebozorgi affirms that this manuscript is an honest, accurate, and transparent account of the study being reported; that no important aspects of the study have been omitted; and that any discrepancies from the study as planned (and, if relevant, registered) have been explained.

## Supporting information

Supplementary Information

## Data Availability

The datasets used and/or analyzed during this study are available from the corresponding author on reasonable request.
