# Supplementary material for: Utilization of health services among the elderly in Iran during the COVID‐19 outbreak: A cross‐sectional study
Source: Health Sci Rep. 2022 Sep 21;5(5):e839. doi: 10.1002/hsr2.839 (PMC9493018; doi:10.1002/hsr2.839)
Supplement: Supplementary file 1 — Supplementary Information [file HSR2-5-0-s001.doc]

**IN THE NAME OF GOD**

**Dear participant**

This questionnaire has been developed aimed at examining the utilization of health services among the elderly during the COVID-19 outbreak. Please answer the questions carefully.

**A)** Demographic and socioeconomic variables

| 1. Age -----years old |
| --- |
| 1. Sex Female ❒ Male ❒ |
| 1. Marital status Single❒ Married❒ |
| 1. Education status Illiterate❒ Primary education (reading and writing) ❒ Diploma & Associate’s degree❒ Bachelor degree & higher ❒ |
| 1. Employment status Employee ❒ Manual worker ❒ Farmer ❒ Self-employed ❒ Retired ❒ Housewife ❒ |
| 1. Habitation status Rural area❒ Urban area❒ |
| 1. Health insurance coverage Yes❒ No❒ |
| 1. Monthly income Less than $100 ❒ $100 to $200 ❒ $200 to $300 ❒ $300 or more ❒ |
| 1. If your answer is ‘Yes’, please select one of the following choices   Social security insurance ❒ Iranian health insurance❒ Health insurance and Rural insurance ❒ Relief foundation insurance❒ Armed forces insurance ❒ Other basic insurances❒ |
| 1. Supplementary insurance coverage Yes❒ No❒ |
| 1. Do you smoke or drink alcohol? Yes❒ No❒ |
| 1. Who do you live with? I am alone ❒ with my spouse ❒ with my wife and children❒ with children ❒ |
| 1. How do you evaluate your health status? Very poor ❒ Poor ❒ Moderate ❒ Good ❒ Very good ❒ |

**14- Morbidity status**

Diabetes Yes❒ No❒ Hypertension Yes❒ No❒

Hyperlipidemia Yes❒ No❒ Cardiovascular diseases Yes❒ No❒

Kidney disease Yes❒ No❒ Cancer Yes❒ No❒

Mental disease Yes❒ No❒ Eye disease Yes❒ No❒

Endocrine and metabolic Yes❒ No❒ Respiratory and infectious Yes❒ No❒

**B) Demand for inpatient services**

15- During the last year, have you been kept under observation for at least 6 hours after the initial examination in the hospital?

Yes❒ No❒ (If your answer is ‘No’, please answer the question of outpatients services)

16) What kind of hospital have you referred to?

Private hospital ❒ Hospital affiliated with social Security ❒

Public hospital affiliated with Ministry of Health ❒ Charity ❒ Public hospital affiliated with other organizations ❒

17- In what way did you refer to the hospital?

Referral by a general practitioner ❒ Referral by a specialist ❒

Referral by a family physician ❒ Referral by other service providers ❒

I referred myself ❒

18- How many times (at least 6 hours) have you been hospitalized during the last year?

……….times, I have been hospitalized.

I have not been hospitalized during the last year ❒

19- What was the source of financing for your inpatient costs to the hospital or medical centers?

Current household income ❒ Health insurance ❒

Use of personal savings ❒ Sale of personal assets ❒ Borrow from others ❒

20- The reason for hospitalization

Diabetes Yes❒ No❒ Hypertension Yes❒ No❒

Hyperlipidemia Yes❒ No❒ Cardiovascular diseases Yes❒ No❒

Kidney disease Yes❒ No❒ Cancer Yes❒ No❒

Mental disease Yes❒ No❒ Eye disease Yes❒ No❒

Endocrine and metabolic Yes❒ No❒ Respiratory and infectious Yes❒ No❒

21- In the last year, did you feel like you needed to be hospitalized, but you refused to be hospitalized?

Yes❒ No❒

**C) Demand for outpatient services**

22- During the last four weeks, have you felt the need to receive outpatient services (doctor's office, midwife's office, laboratory, clinic, photography, etc.)?

Yes❒ No❒ (If your answer is ‘No’, please don't answer the following questions)

23- Have you been referred to health care centers for outpatient services during the past four weeks?

Yes❒ No❒

24- How many times have you referred to health care centers to receive the service during the past four weeks?

……….times, I have been hospitalized.

I have not been hospitalized during the last year ❒

25- What was the source of financing for you/ your family members for outpatient costs to the hospital or medical centers?

Current household income ❒ Health insurance ❒

Use of personal savings ❒ Sale of personal assets ❒ Borrow from others ❒

26- The reason for outpatient

Diabetes Yes❒ No❒ Hypertension Yes❒ No❒

Hyperlipidemia Yes❒ No❒ Cardiovascular diseases Yes❒ No❒

Kidney disease Yes❒ No❒ Cancer Yes❒ No❒

Mental disease Yes❒ No❒ Eye disease Yes❒ No❒

Endocrine and metabolic Yes❒ No❒ Respiratory and infectious Yes❒ No❒
